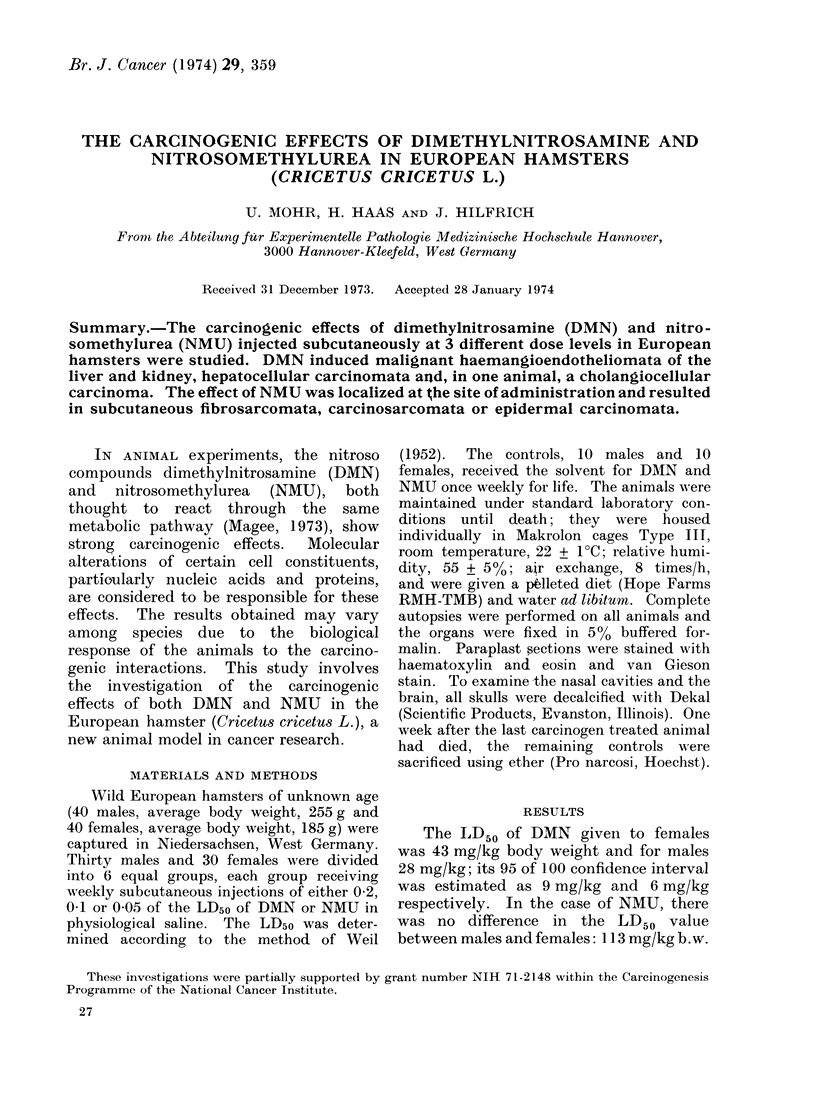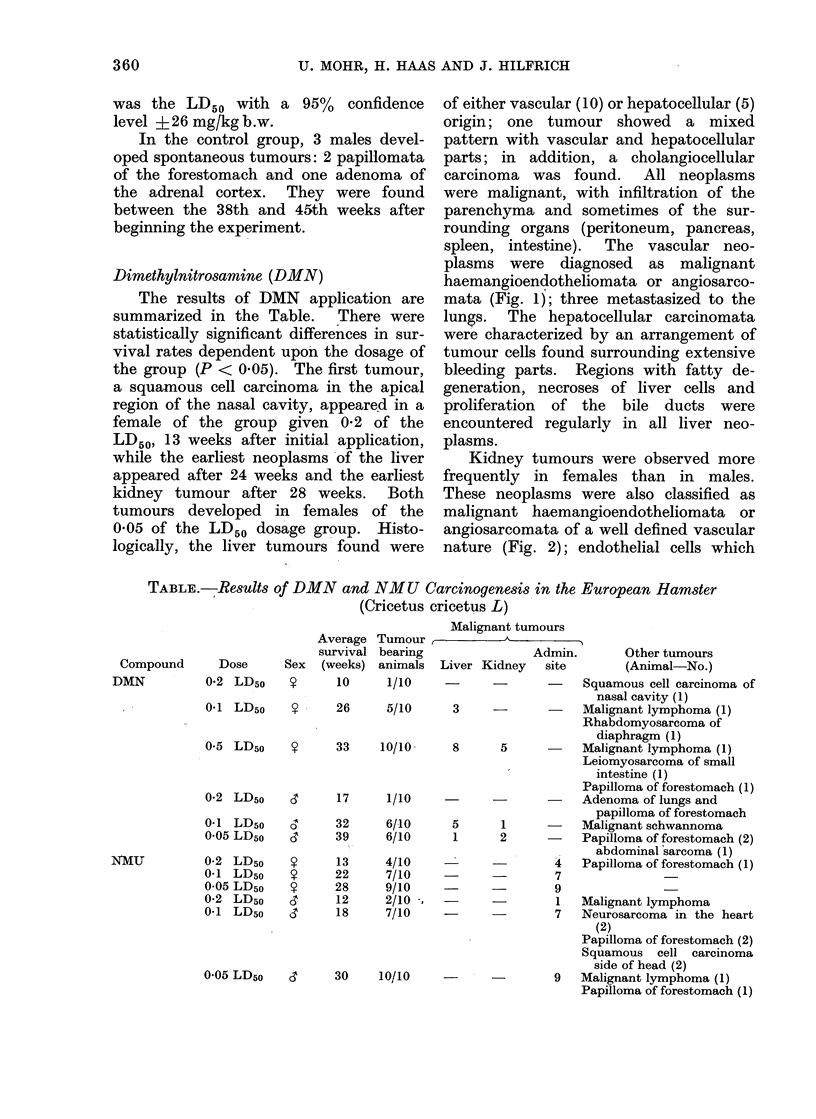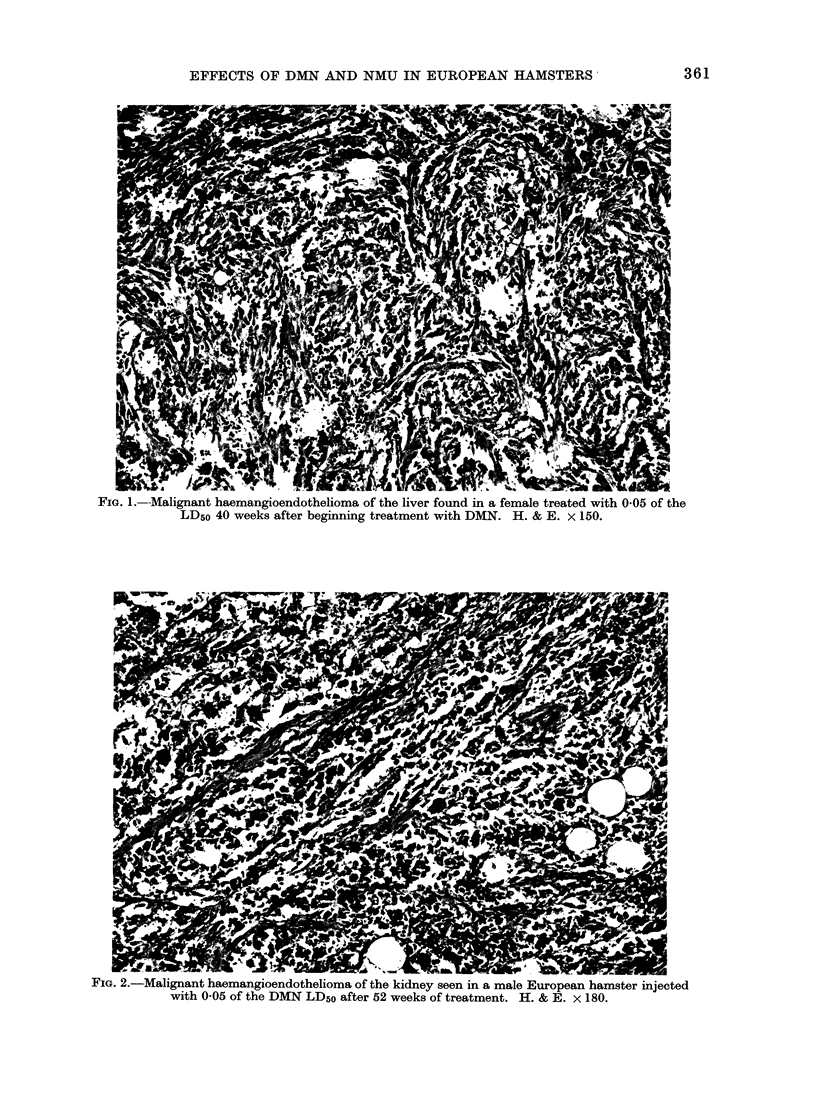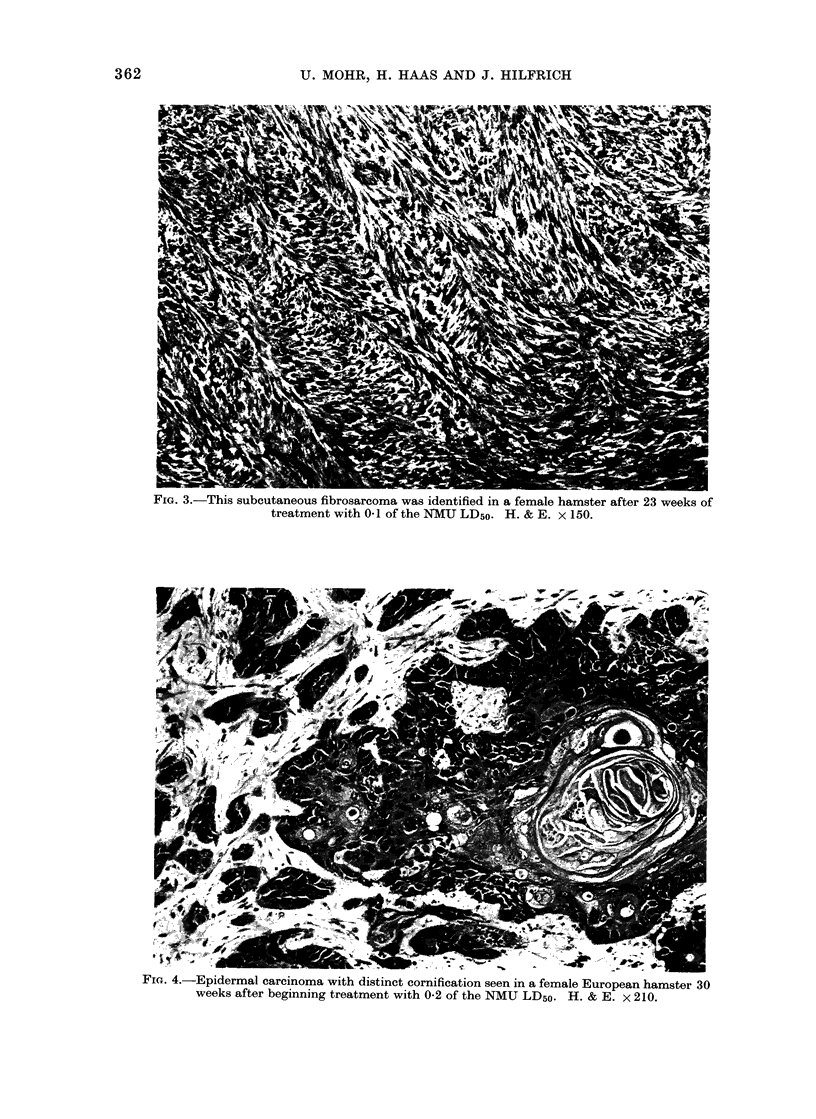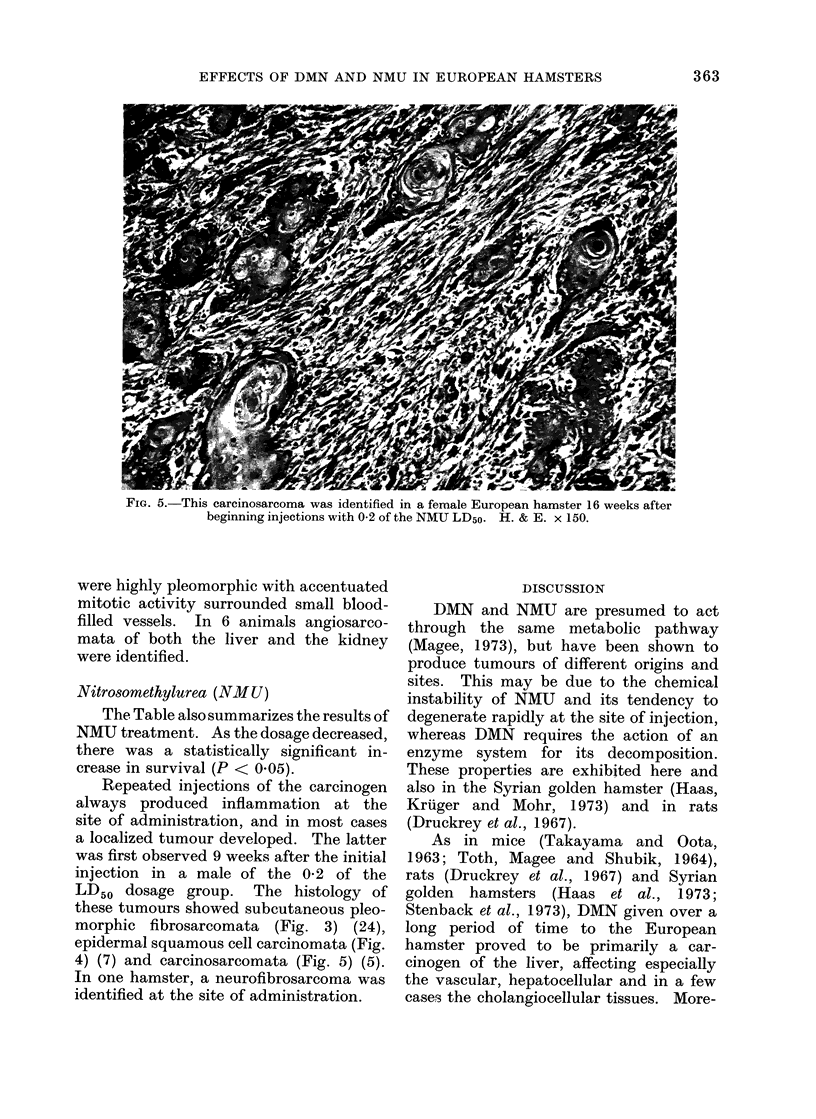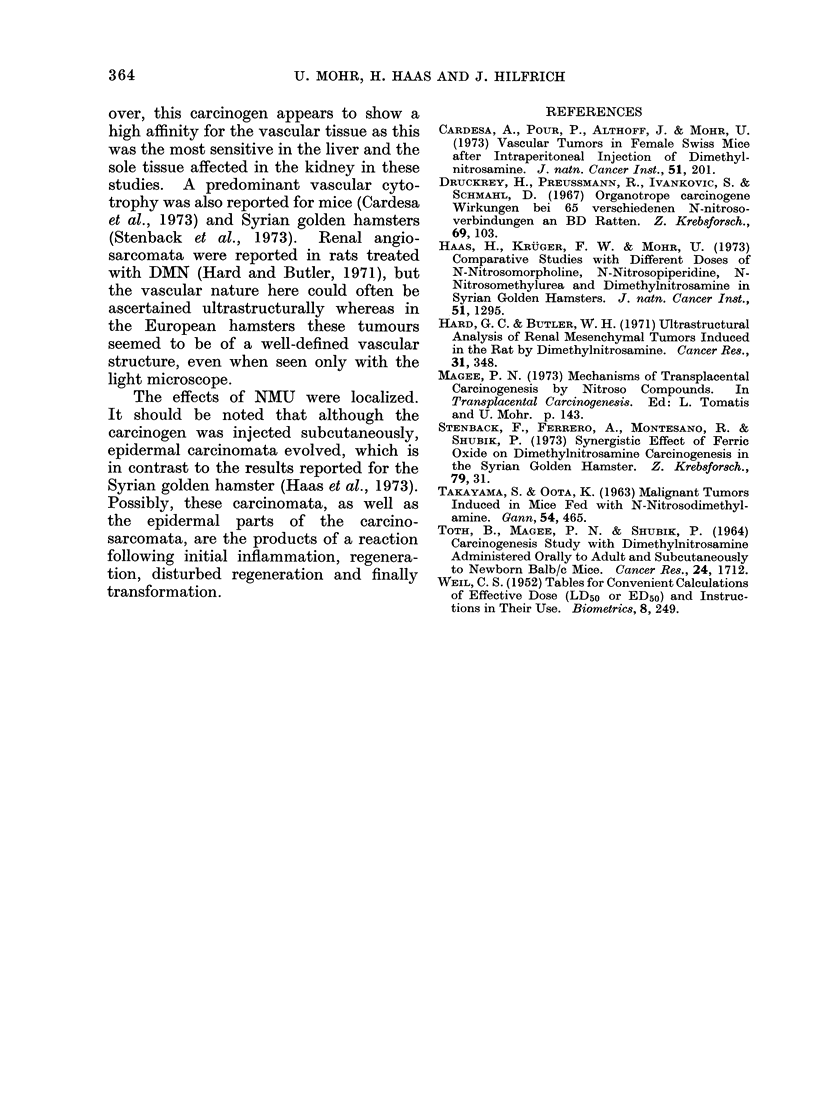# The Carcinogenic Effects of Dimethylnitrosamine and Nitrosomethylurea in European Hamsters (Cricetus cricetus L.)

**DOI:** 10.1038/bjc.1974.83

**Published:** 1974-05

**Authors:** U. Mohr, H. Haas, J. Hilfrich

## Abstract

**Images:**


*Br. J. Cancer* (1974) **29**, 359

### THE CARCINOGENIC EFFECTS OF DIMETHYLNITROSAMINE AND NITROSOMETHYLUREA IN EUROPEAN HAMSTERS (*CRICETUS CRICETUS* L.)

U. MOHR, H. HAAS AND J. HILFRICH

*From the Abteilung für Experimentelle Pathologie Medizinische Hochschule Hannover, 3000 Hannover-Kleefeld, West Germany*

Received 31 December 1973. Accepted 28 January 1974

**Summary.—The carcinogenic effects of dimethylnitrosamine (DMN) and nitrosomethylurea (NMU) injected subcutaneously at 3 different dose levels in European hamsters were studied. DMN induced malignant haemangioendotheliomata of the liver and kidney, hepatocellular carcinomata and, in one animal, a cholangiocellular carcinoma. The effect of NMU was localized at the site of administration and resulted in subcutaneous fibrosarcomata, carcinosarcomata or epidermal carcinomata.**

IN ANIMAL experiments, the nitroso compounds dimethylnitrosamine (DMN) and nitrosomethylurea (NMU), both thought to react through the same metabolic pathway (Magee, 1973), show strong carcinogenic effects. Molecular alterations of certain cell constituents, particularly nucleic acids and proteins, are considered to be responsible for these effects. The results obtained may vary among species due to the biological response of the animals to the carcinogenic interactions. This study involves the investigation of the carcinogenic effects of both DMN and NMU in the European hamster (*Cricetus cricetus L.*), a new animal model in cancer research.

### MATERIALS AND METHODS

Wild European hamsters of unknown age (40 males, average body weight, 255 g and 40 females, average body weight, 185 g) were captured in Niedersachsen, West Germany. Thirty males and 30 females were divided into 6 equal groups, each group receiving weekly subcutaneous injections of either 0·2, 0·1 or 0·05 of the $LD_{50}$ of DMN or NMU in physiological saline. The $LD_{50}$ was determined according to the method of Weil (1952). The controls, 10 males and 10 females, received the solvent for DMN and NMU once weekly for life. The animals were maintained under standard laboratory conditions until death; they were housed individually in Makrolon cages Type III, room temperature, 22 ± 1°C; relative humidity, 55 ± 5%; air exchange, 8 times/h, and were given a pelleted diet (Hope Farms RMH-TMB) and water *ad libitum*. Complete autopsies were performed on all animals and the organs were fixed in 5% buffered formalin. Paraplast sections were stained with haematoxylin and eosin and van Gieson stain. To examine the nasal cavities and the brain, all skulls were decalcified with Dekal (Scientific Products, Evanston, Illinois). One week after the last carcinogen treated animal had died, the remaining controls were sacrificed using ether (Pro narcosi, Hoechst).

### RESULTS

The $LD_{50}$ of DMN given to females was 43 mg/kg body weight and for males 28 mg/kg; its 95 of 100 confidence interval was estimated as 9 mg/kg and 6 mg/kg respectively. In the case of NMU, there was no difference in the $LD_{50}$ value between males and females: 113 mg/kg b.w.

These investigations were partially supported by grant number NIH 71-2148 within the Carcinogenesis Programme of the National Cancer Institute.

was the $LD_{50}$ with a 95% confidence level $\pm 26$ mg/kg b.w.

In the control group, 3 males developed spontaneous tumours: 2 papillomata of the forestomach and one adenoma of the adrenal cortex. They were found between the 38th and 45th weeks after beginning the experiment.

*Dimethylnitrosamine (DMN)*

The results of DMN application are summarized in the Table. There were statistically significant differences in survival rates dependent upon the dosage of the group ($P < 0{\cdot}05$). The first tumour, a squamous cell carcinoma in the apical region of the nasal cavity, appeared in a female of the group given 0·2 of the $LD_{50}$, 13 weeks after initial application, while the earliest neoplasms of the liver appeared after 24 weeks and the earliest kidney tumour after 28 weeks. Both tumours developed in females of the 0·05 of the $LD_{50}$ dosage group. Histologically, the liver tumours found were of either vascular (10) or hepatocellular (5) origin; one tumour showed a mixed pattern with vascular and hepatocellular parts; in addition, a cholangiocellular carcinoma was found. All neoplasms were malignant, with infiltration of the parenchyma and sometimes of the surrounding organs (peritoneum, pancreas, spleen, intestine). The vascular neoplasms were diagnosed as malignant haemangioendotheliomata or angiosarcomata (Fig. 1); three metastasized to the lungs. The hepatocellular carcinomata were characterized by an arrangement of tumour cells found surrounding extensive bleeding parts. Regions with fatty degeneration, necroses of liver cells and proliferation of the bile ducts were encountered regularly in all liver neoplasms.

Kidney tumours were observed more frequently in females than in males. These neoplasms were also classified as malignant haemangioendotheliomata or angiosarcomata of a well defined vascular nature (Fig. 2); endothelial cells which

TABLE.—*Results of DMN and NMU Carcinogenesis in the European Hamster* (Cricetus cricetus *L*)

| Compound | Dose | Sex | Average survival (weeks) | Tumour bearing animals | Malignant tumours: Liver | Malignant tumours: Kidney | Malignant tumours: Admin. site | Other tumours (Animal—No.) |
|---|---|---|---|---|---|---|---|---|
| DMN | 0·2 $LD_{50}$ | ♀ | 10 | 1/10 | — | — | — | Squamous cell carcinoma of nasal cavity (1) |
| | 0·1 $LD_{50}$ | ♀ | 26 | 5/10 | 3 | — | — | Malignant lymphoma (1) Rhabdomyosarcoma of diaphragm (1) |
| | 0·5 $LD_{50}$ | ♀ | 33 | 10/10 | 8 | 5 | — | Malignant lymphoma (1) Leiomyosarcoma of small intestine (1) Papilloma of forestomach (1) |
| | 0·2 $LD_{50}$ | ♂ | 17 | 1/10 | — | — | — | Adenoma of lungs and papilloma of forestomach |
| | 0·1 $LD_{50}$ | ♂ | 32 | 6/10 | 5 | 1 | — | Malignant schwannoma |
| | 0·05 $LD_{50}$ | ♂ | 39 | 6/10 | 1 | 2 | — | Papilloma of forestomach (2) abdominal sarcoma (1) |
| NMU | 0·2 $LD_{50}$ | ♀ | 13 | 4/10 | — | — | 4 | Papilloma of forestomach (1) |
| | 0·1 $LD_{50}$ | ♀ | 22 | 7/10 | — | — | 7 | — |
| | 0·05 $LD_{50}$ | ♀ | 28 | 9/10 | — | — | 9 | — |
| | 0·2 $LD_{50}$ | ♂ | 12 | 2/10 | — | — | 1 | Malignant lymphoma |
| | 0·1 $LD_{50}$ | ♂ | 18 | 7/10 | — | — | 7 | Neurosarcoma in the heart (2) Papilloma of forestomach (2) Squamous cell carcinoma side of head (2) |
| | 0·05 $LD_{50}$ | ♂ | 30 | 10/10 | — | — | 9 | Malignant lymphoma (1) Papilloma of forestomach (1) |

FIG. 1.—Malignant haemangioendothelioma of the liver found in a female treated with 0·05 of the $LD_{50}$ 40 weeks after beginning treatment with DMN. H. & E. ×150.

FIG. 2.—Malignant haemangioendothelioma of the kidney seen in a male European hamster injected with 0·05 of the DMN $LD_{50}$ after 52 weeks of treatment. H. & E. ×180.

FIG. 3.—This subcutaneous fibrosarcoma was identified in a female hamster after 23 weeks of treatment with 0·1 of the NMU $LD_{50}$. H. & E. ×150.

FIG. 4.—Epidermal carcinoma with distinct cornification seen in a female European hamster 30 weeks after beginning treatment with 0·2 of the NMU $LD_{50}$. H. & E. ×210.

FIG. 5.—This carcinosarcoma was identified in a female European hamster 16 weeks after beginning injections with 0·2 of the NMU $LD_{50}$. H. & E. × 150.

were highly pleomorphic with accentuated mitotic activity surrounded small blood-filled vessels. In 6 animals angiosarcomata of both the liver and the kidney were identified.

*Nitrosomethylurea* (*NMU*)

The Table also summarizes the results of NMU treatment. As the dosage decreased, there was a statistically significant increase in survival ($P < 0{\cdot}05$).

Repeated injections of the carcinogen always produced inflammation at the site of administration, and in most cases a localized tumour developed. The latter was first observed 9 weeks after the initial injection in a male of the 0·2 of the $LD_{50}$ dosage group. The histology of these tumours showed subcutaneous pleomorphic fibrosarcomata (Fig. 3) (24), epidermal squamous cell carcinomata (Fig. 4) (7) and carcinosarcomata (Fig. 5) (5). In one hamster, a neurofibrosarcoma was identified at the site of administration.

### DISCUSSION

DMN and NMU are presumed to act through the same metabolic pathway (Magee, 1973), but have been shown to produce tumours of different origins and sites. This may be due to the chemical instability of NMU and its tendency to degenerate rapidly at the site of injection, whereas DMN requires the action of an enzyme system for its decomposition. These properties are exhibited here and also in the Syrian golden hamster (Haas, Krüger and Mohr, 1973) and in rats (Druckrey *et al.*, 1967).

As in mice (Takayama and Oota, 1963; Toth, Magee and Shubik, 1964), rats (Druckrey *et al.*, 1967) and Syrian golden hamsters (Haas *et al.*, 1973; Stenback *et al.*, 1973), DMN given over a long period of time to the European hamster proved to be primarily a carcinogen of the liver, affecting especially the vascular, hepatocellular and in a few cases the cholangiocellular tissues. Moreover, this carcinogen appears to show a high affinity for the vascular tissue as this was the most sensitive in the liver and the sole tissue affected in the kidney in these studies. A predominant vascular cytotrophy was also reported for mice (Cardesa *et al.*, 1973) and Syrian golden hamsters (Stenback *et al.*, 1973). Renal angiosarcomata were reported in rats treated with DMN (Hard and Butler, 1971), but the vascular nature here could often be ascertained ultrastructurally whereas in the European hamsters these tumours seemed to be of a well-defined vascular structure, even when seen only with the light microscope.

The effects of NMU were localized. It should be noted that although the carcinogen was injected subcutaneously, epidermal carcinomata evolved, which is in contrast to the results reported for the Syrian golden hamster (Haas *et al.*, 1973). Possibly, these carcinomata, as well as the epidermal parts of the carcinosarcomata, are the products of a reaction following initial inflammation, regeneration, disturbed regeneration and finally transformation.

### REFERENCES

Cardesa, A., Pour, P., Althoff, J. & Mohr, U. (1973) Vascular Tumors in Female Swiss Mice after Intraperitoneal Injection of Dimethylnitrosamine. *J. natn. Cancer Inst.*, **51**, 201.

Druckrey, H., Preussmann, R., Ivankovic, S. & Schmahl, D. (1967) Organotrope carcinogene Wirkungen bei 65 verschiedenen N-nitrosoverbindungen an BD Ratten. *Z. Krebsforsch.*, **69**, 103.

Haas, H., Krüger, F. W. & Mohr, U. (1973) Comparative Studies with Different Doses of N-Nitrosomorpholine, N-Nitrosopiperidine, N-Nitrosomethylurea and Dimethylnitrosamine in Syrian Golden Hamsters. *J. natn. Cancer Inst.*, **51**, 1295.

Hard, G. C. & Butler, W. H. (1971) Ultrastructural Analysis of Renal Mesenchymal Tumors Induced in the Rat by Dimethylnitrosamine. *Cancer Res.*, **31**, 348.

Magee, P. N. (1973) Mechanisms of Transplacental Carcinogenesis by Nitroso Compounds. In *Transplacental Carcinogenesis*. Ed: L. Tomatis and U. Mohr. p. 143.

Stenback, F., Ferrero, A., Montesano, R. & Shubik, P. (1973) Synergistic Effect of Ferric Oxide on Dimethylnitrosamine Carcinogenesis in the Syrian Golden Hamster. *Z. Krebsforsch.*, **79**, 31.

Takayama, S. & Oota, K. (1963) Malignant Tumors Induced in Mice Fed with N-Nitrosodimethylamine. *Gann*, **54**, 465.

Toth, B., Magee, P. N. & Shubik, P. (1964) Carcinogenesis Study with Dimethylnitrosamine Administered Orally to Adult and Subcutaneously to Newborn Balb/c Mice. *Cancer Res.*, **24**, 1712.

Weil, C. S. (1952) Tables for Convenient Calculations of Effective Dose ($LD_{50}$ or $ED_{50}$) and Instructions in Their Use. *Biometrics*, **8**, 249.